# ‘Moving People up the Sympathy Spectrum’: Building Hypotheses About How Abortion Values Clarification and Attitude Transformation (VCAT) Can Change Healthcare Provider Behaviours

**DOI:** 10.1111/hex.70841

**Published:** 2026-08-22

**Authors:** Shelly Makleff, Carrie Van Rensburg, Bronwen Merner, Alexandra Wollum, Louise Keogh

**Affiliations:** ^1^ Centre for Health Equity University of Melbourne Parkville VIC Australia; ^2^ Ibis Reproductive Health Oakland CA USA

**Keywords:** abortion stigma, behaviour change, empathy, healthcare settings, person‐centred abortion care, provider stigma, values clarification

## Abstract

**Background:**

Abortion stigma limits access to person‐centred abortion care globally. Values clarification and attitude transformation (VCAT) is widely used to address abortion stigma and recommended by the World Health Organization to train abortion providers. Yet evidence is lacking about whether and how VCAT influences healthcare providers' behaviours. This study aims to generate hypotheses regarding (a) the specific behaviours that VCAT might influence and (b) the mechanisms of change.

**Methods:**

This qualitative study generated hypotheses based on interviews with people who facilitated, designed and/or participated in VCAT globally. We recruited via email and by posting on online communities. Study design was informed by two behaviour change frameworks and engaged the concepts of stigma and person‐centred care. We conducted semi‐structured interviews online and analysed data thematically to develop hypotheses about VCAT's influence on behaviours.

**Results:**

The 17 interviewees (15 facilitators/curriculum designers, 2 VCAT participants) worked in five world regions. They described five categories of person‐centred abortion care behaviours that VCAT can facilitate: increasing abortion‐related communication with colleagues; reducing delays and obstruction of abortion care; enabling abortion seekers to access abortion care; expanding own abortion provision; and engaging in high‐quality, non‐judgemental abortion care. Interviewees described distinct forms of empathy through which VCAT influenced behaviour.

**Discussion:**

This study helps fill an evidence gap by offering hypotheses about how abortion VCAT can lead to behaviour change and which behaviours VCAT might influence in healthcare providers. We illustrate VCAT's ability to build empathy towards and destigmatise abortion across sociocultural contexts, forms of stigma, and legal frameworks – potentially motivating behaviour change along a spectrum from opposing to supporting abortion access. These hypotheses can be tested in future evaluations to assess whether VCAT contributes to change in a range of person‐centred care behaviours. Our findings about VCAT's strengths and its potential gaps can inform future work to refine VCAT and develop complementary strategies to contribute to person‐centred abortion care provision across contexts.

**Patient or Public Contribution:**

One of the co‐investigators has lived experience implementing and designing values clarification trainings (the focus of this paper). Prior studies engaging people with lived experience of abortion care informed the design of this study. Patients, service users, care‐givers, people with lived experience or members of the public were not involved in the conduct of study, analysis or interpretation of the data or in preparation of the manuscript.

## 1 Introduction

Access to high‐quality abortion care is a recognised, fundamental human right [1]. A key dimension of high‐quality care is person‐centredness, defined by the World Health Organization (WHO) as care that responds to patient preferences, needs and values and empowers them to take charge of their own health [2, 3]. In practice, however, a 2018 review found that most abortion healthcare services worldwide fail to provide person‐centred care, reflecting system‐level issues including medical misogyny and social stigma underpinned by gendered social norms [4].

Abortion stigma is a gendered social phenomenon that frames abortion as morally wrong and socially unacceptable [5, 6, 7]. It operates at multiple interrelated levels: structural (e.g., policies, laws, health systems with arduous requirements to receive abortion), community (e.g., interpersonal interactions that judge or punish abortion seekers and providers), and individual (e.g., abortion seekers feel guilty or anticipate being judged) [8, 9, 10]. This paper focuses on a form of interpersonal stigma – the stigmatising behaviours enacted by people working in healthcare towards abortion seekers, reflecting the role of the healthcare workforce in perpetuating abortion stigma [9, 11, 12]. Because abortion stigma is increasingly understood by scholars as a ‘socio‐cultural process […] upon which power relations are produced and legitimated’ [13], the power differential between healthcare providers and their patients [14] plays an important role in how stigma is produced in healthcare interactions. A 2022 scoping review identified forms of stigma enacted by health workers, including denying or obstructing care and (adverently or inadvertently) judging, mistreating, or threatening abortion seekers [9]. Such stigmatising behaviours, which undermine person‐centred care, have been documented in a range of legal contexts, from decriminalised to highly restricted abortion access [12, 15, 16, 17, 18]. Abortion seekers subjected to these behaviours can experience distress and delayed care, with negative economic and psychological consequences [19, 20]. Past experiences of stigma at abortion care facilities may also result in abortion seekers avoiding further interactions with the health system, as they may anticipate judgement or poor treatment from formal health services [21, 22]. As such, reducing abortion stigma in service delivery is a global public health imperative that can promote the accessibility and person‐centeredness of abortion care [23].

Despite a consensus about the urgency of reducing stigmatising behaviours in abortion care, scoping and systematic reviews highlight a dearth of effective interventions [9, 24, 25, 26]. Evaluations of sexual and reproductive health stigma‐reduction strategies among health workers focus on attitudes, knowledge, or intended behaviours, but few have measured changes in behaviour [25, 26]. As such, while diverse efforts to reduce stigma are being implemented [25], there is little evidence about how to effectively change behaviour in ways that improve the accessibility and person‐centredness of abortion care.

Values clarification and attitude transformation (VCAT) is one type of intervention that has been widely used by non‐governmental organisations and health services to address abortion stigma [27, 28]. The approach, which emerged from the field of humanistic psychology in the 1960s, was later adapted for use around stigmatised sexual and reproductive health (SRH) topics [29]. VCAT promotes reflection on values, which are ‘guiding principles in life’; values can influence attitudes, which are specific judgements on a topic, and in turn guide behaviour [30]. VCAT is recommended by the World Health Organization for training abortion providers [31], and a range of medical education and service delivery organisations have adapted and implemented this approach, which they may encourage or require for their healthcare providers [32, 33]. Based on work in South Africa in the early 2000s, SRH organisation Ipas developed an abortion VCAT training manual that the organisation has updated, adapted and implemented in more than 30 countries [27, 34]. Values clarification is also used by a range of organisations for other stigmatised SRH topics including HIV [35, 36] and for harmful practices related to gender and social norms, including female genital mutilation [37].

The abortion VCAT approach comprises a series of interrelated group activities for healthcare providers, policy makers, and/or community members. It is typically held over multiple days, and led by well‐trained facilitators, some of whom may be healthcare providers. The workshops seek to generate self‐reflection about abortion‐related values and behaviours, deepen understanding of and build empathy towards abortion seekers, and support participants to consider their professional responsibilities towards abortion seekers [27, 34, 38]. The theoretical framework for the Ipas manual, based on Ajzen's Theory of Planned Behaviour [39], articulates a multi‐stage process in which participants choose, value, and act on their abortion‐related values [27, 29, 34]. As the 2023 Ipas manual states, ‘values clarification is a process that helps ensure that choices and actions are the result of informed, reasoned thoughts and feelings’ [34]. The theorical framework diagram in the manual shows a pathway from ‘attitude transformation’ to ‘behavioural intention’ to ‘performance’ [34]. However, the types of performance expected after VCAT are not specified, and the pathway from attitudes or behavioural intentions to actual behaviour is not explained in the theoretical framework (perhaps reflecting lack of evidence on this topic), leaving a gap in theory about whether and how VCAT may influence behaviour change.

Mirroring this gap in conceptualisation, we lack empirical evidence that VCAT successfully influences behaviours [25, 26, 28]. For example, a 12‐country study of VCAT in Asia, Africa and Latin America assessed knowledge, attitudes (including those related to stigma), and intended behaviours before and after the intervention using pre‐post surveys, but did not seek to gauge behaviour change [27]. Similarly, two studies of VCAT with US‐based medical trainees examined attitudes and intended behaviours, but did not measure any impact of VCAT on behaviours [38, 40]. A pre‐post survey of VCAT implementation in Pakistan measured attitudes and knowledge but not behavioural intentions or behaviours; findings from in‐depth interviews suggest some VCAT participants felt an increased sense of professional responsibility to provide abortion care or may have improved their referrals, service provision, or communication with abortion patients [41]. The only published longitudinal study of VCAT, conducted in Ethiopia, quantitatively measured service volume following VCAT as a proxy for behaviour change – finding a temporary increase in service volume that was not sustained after 10 months [28]. Yet the study's qualitative findings suggested that some VCAT participants may have started providing abortion to a wider range of clients or modified their gatekeeping behaviours [28]. Taken together, these evaluations, primarily in settings where abortion is legally restricted, result in an evidence base with little examination of VCAT's role in behaviour change. Further, VCAT evaluations have focused on implementation of a particular VCAT manual in a single organisation or context, without examining the common aspects that might make VCAT effective across settings. Given the critical role of healthcare providers as potential enactors of stigmatising behaviours, and the dearth of effective interventions, more evidence is needed to understand what behaviours VCAT might change in healthcare providers in different settings, whether VCAT can change these behaviours, and if so, through what mechanisms.

VCAT is widely implemented despite persistent theory and evidence gaps about whether and how it influences behaviour. As such, this study aims to generate hypotheses regarding (a) the specific behaviours that abortion VCAT can influence in healthcare providers, and (b) the mechanisms through which VCAT might influence these behaviours, in a range of socio‐cultural, clinical, and legal contexts. Findings can help researchers identify what behaviours and pathways to behaviour change can be measured in future VCAT evaluations, and as such can inform future efforts to assess whether VCAT generates behaviour change in health care providers.

## 2 Methods

### 2.1 Study Design

This qualitative study used in‐depth interviews with people experienced in abortion VCAT (primarily facilitators and curriculum designers, as well as healthcare providers who had participated in VCAT, anywhere in the world) to build hypotheses about which health provider behaviours VCAT may influence and through what mechanisms. The hypothesis development approach drew on theory‐informed reflections about how VCAT works among curriculum designers and facilitators, as well as their secondhand observations about how VCAT influenced healthcare provider participants in VCAT workshops they had facilitated; the process also engaged firsthand accounts from the few healthcare provider VCAT participants in the study, which we used to add new insights or corroborate the observations made by the curriculum designers and facilitators. As such, this study is not an evaluation of VCAT but rather a hypothesis development study that is a necessary precendent to future studies seeking to measure changes in specific behaviours among healthcare providers who have participated in VCAT.

Our study design, data collection, analysis and interpretation were informed by two interrelated behaviour change research frameworks. First, the COM‐B framework, which posits that a target behaviour (B) is influenced by someone's capability (C), opportunity (O), and motivation (M) to carry out the behaviour [42]. Second, the action, actor, context, target, time (AACTT) framework [43], which we used to explicitly identify specific behaviours (B) that might be influenced by VCAT. Identifying specific target behaviours is a critical step before designing and evaluating interventions, yet is often overlooked [43].

### 2.2 Participants and Recruitment

Participants were eligible if they had designed and/or facilitated VCAT workshops for healthcare providers in the last 10 years, or if they were healthcare providers who had participated in a VCAT workshop in the last 3 years, anywhere in the world, and could comfortably participate in an English‐language interview. Recruitment was carried out by posting on a closed‐member online global community of abortion stigma activists, professionals, and researchers, and through personalised email invitations to non‐governmental organisations (NGOs) and clinics known to implement VCAT. Anyone receiving an invitation was asked to share it with other relevant potential participants. Through snowballing, our recruitment materials were shared with a global community of practice for VCAT facilitators and within large international NGOs. This yielded many expressions of interest from facilitators and curriculum designers but few from healthcare provider VCAT participants. Recruitment materials included a link to an online expression of interest survey (Qualtrics) gathering information about their country or region of work, profession, and VCAT experience. From the 45 expressions of interest received with complete data, we selected a sample to ensure maximum variation [44] in location, professional training, and type of VCAT experience. Because one‐third of the expressions of interest came from two international NGOs, we invited no more than four participants per organisation to avoid overrepresenting any one VCAT model and sought a diverse set of organisations and perspectives in our sample. The expression of interest survey did not collect data about gender so that characteristic was not incorporated into the maximum variation sampling strategy.

### 2.3 Data Collection

The first and last authors developed a semi‐structured interview guide asking about participants' experiences of VCAT, perceptions of its goals, what they believed it was best at achieving, how VCAT might address stigma and person‐centred care, and what behaviours it might influence in healthcare providers. The interview guide probed on elements of the COM‐B framework for behaviour change (capability, opportunity, motivation) [42] and asked participants to be as specific as possible when describing behaviours, in line with the AACTT framework [43]. Participants were asked to describe behaviour change they had experienced themselves or had observed in others, and to hypothesise about which other behaviours VCAT might influence and the potential pathways to behaviour change. The guide included different prompts for VCAT participants and facilitators to elicit information from these different perspectives.

Invited participants signed a consent form in advance of a Zoom interview scheduled at a time of their preference. At the beginning of each interview, the interviewer informed interviewees that they had never facilitated or designed VCAT and were not affiliated with any VCAT‐implementing organisation, and that the study intended to develop hypotheses about how VCAT influenced behaviour rather than evaluate VCAT's effects. All interviews were conducted in English by the first author, audio‐recorded on Zoom, and transcribed using Zoom's audio transcription function, with participant consent. Participants received an e‐gift card (equivalent to $30 AUD) after completing the interview. The study was approved by the University of Melbourne Human Research Ethics Board (ID 30693).

### 2.4 Data Analysis

To familiarise with the data, author 2 compared the interview audio recordings with the automatically generated transcripts and corrected the transcripts if needed. Using the concepts of COM‐B, person‐centred care, and stigma, and based on our familiarity with the data, authors 1 and 2 developed a preliminary codebook for thematic analysis [45]. They then coded the same three transcripts, compared their coding, and adjusted the codebook. The second author coded all additional transcripts, discussing the process in weekly meetings with author 1. Once all transcripts were coded, authors 1 and 2 refined the focus to the codes describing behaviour change (COM‐B; person‐centred care; what VCAT is most useful for; change over time due to VCAT; shifts in attitudes, behaviour and knowledge). Within excerpts coded as behaviours, we reviewed all excerpts to generate examples of the specific types of behaviours that participants hypothesised VCAT might influence. We used the AACTT (action, actor, context, target, time) framework [43] to inform our conceptualisation of these behaviours, for example by specifying who engaged in the behaviour (actor; action), at what point in the health service (time; context), and towards whom (target). The behaviours identified in this analysis are presented in Section 3.2. We then wrote summaries of the key themes in all behaviour‐related codes, with a focus on the mechanisms through which participants believed behaviours are influenced by VCAT (Section 3.3). Reflecting the sample, hypotheses about behaviours and mechanisms of change presented in this paper are based primarily on interviews with facilitators, with VCAT participants' narratives used to corroborate or enrich the findings. All quotes are presented using participant identifiers to protect confidentiality.

## 3 Results

### 3.1 Participants

The 17 participants had VCAT experience in five regions of the world. Some worked in one country (e.g., Australia, United States, Nepal, Ghana, Sierra Leone, Kenya), others conducted VCAT across one or multiple regions of the world. Three participants were male, 14 were female (Table 1).

**Table 1:** Participant characteristics (*n* = 17).

Participant characteristics	*n* = 17
*Age range at time of interview*	
18–24 years old	0 (0%)
25–39 years old	7 (41%)
40+ years old	8 (47%)
Not specified	2 (12%)
*Regions where participants experienced VCAT*	
East Asia and Pacific (all Australia)	5 (29%)
Multi‐region	4 (23%)
Sub‐Saharan Africa	3 (18%)
North America (all USA)	3 (18%)
South Asia	1 (6%)
Latin America and Caribbean	1 (6%)
*Gender*	
Female	14 (82%)
Male	3 (18%)
*Time working in abortion sector*	
< 10 years	6 (35%)
10–20 years	8 (47%)
> 20 years	1 (6%)
Not specified	2 (12%)
*Type of VCAT experience*	
Facilitator only	7 (41%)
Healthcare provider	2
Not healthcare provider	5
Facilitator‐participant	4 (23%)
Healthcare provider	3
Not healthcare provider	1
Facilitator‐curriculum designer	4 (23%)
Healthcare provider	2
Not healthcare provider	2
Participant only	2 (12%)
Healthcare provider	2
Not healthcare provider	0
*Participant workplace* (*n* = 18)^a^	
Local healthcare service delivery	5 (29%)
International non‐governmental organisation (INGO)	6 (35%)
Community organisation	5 (29%)
Medical education and training	2 (12%)

^a^ *n* = 18 and the total is > 100% because one participant conducted VCAT for two different workplaces.

Participants had a range of VCAT experiences that informed their perceptions and description of VCAT. Fifteen of 17 had facilitated VCAT. Of these, seven were facilitators only (‘facilitator’), some of whom described ongoing engagement with a group of participants in the same organisation, allowing them to observe behaviour beyond the scope of one VCAT workshop. Four were facilitators who had also participated in VCAT in the past (‘facilitator‐participant’), and spoke to both perspectives in the interview. Four were facilitators who had also designed VCAT curricula (‘facilitator‐curriculum designer’); they had developed VCAT exercises for medical students or about stigmatised groups who might seek abortion care, and were grounded in the theory underpinning VCAT practice. The two who had never been facilitators were healthcare providers who had each participated in one VCAT workshop/activity (‘participant’). Within each category, some interviewees were trained as healthcare providers (e.g., doctor, nurse, social worker), as shown in Table 1. These various perspectives are woven together in the subsequent sections to build hypotheses about how VCAT can influence healthcare provider behaviours.

Interviewees described experiences with a range of VCAT modalities (both in‐person and online), including: implementation of multi‐day VCAT workshops for healthcare teams across all levels of staff (executives, doctors, nurses, administrators); a full day workshop for government officials; short (one hour to half‐day) VCAT activities in local clinics, hospitals, or non‐profits; implementation of a VCAT workshop as part of quality improvement programmes; and one‐on‐one or small group VCAT sessions with medical students.

### 3.2 Hypothesis Development: What Behaviours Might VCAT Shift in Healthcare Providers?

Interviewees described a range of behaviours related to person‐centred abortion care that they hypothesised VCAT could facilitate. These fall into five main categories: (a) increasing abortion‐related communication with colleagues; (b) reducing delays and obstruction of abortion care; (c) enabling abortion seekers to access abortion care; (d) expanding own abortion provision; and (e) engaging in high‐quality, non‐judgemental abortion care. These behaviours, which both directly and indirectly reflect abortion stigma, are summarised in Table 2 and described in detail with example quotes.

**Table 2:** Specific behaviours that VCAT is hypothesised to influence among healthcare providers to facilitate person‐centred abortion care.

Category of behaviour	Description of behaviours within each category
A: Increasing abortion‐related communication with colleagues	Talking explicitly about abortion with colleagues in the workplace Communicating about one's needs and preferences in relation to abortion provision with colleagues
B: Reducing delays and obstruction of abortion care	Refraining from reporting alleged abortion seekers to authorities in criminalised contexts Reducing intentional delays of abortion care Respecting patients’ rights to access care (e.g. actively providing information)
C: Enabling abortion seekers to access abortion care	Proactively seeking creative ways to ensure access for patients (e.g., finding pathways to care) without directly providing abortion care Finding ways to enable care (e.g., set up exam rooms) and tolerate discomfort in accordance with their individual beliefs to support patient rights, without directly providing abortion care Starting to refer for abortion care
D: Expanding own abortion provision	Starting to provide abortion care when had not done so before Providing abortions at later gestational stages than before Providing more stigmatised forms of abortion care (e.g., to abortion seekers who have had more than one abortion)
E: Engaging in high‐quality, non‐judgemental abortion care provision	Engaging patients with compassion, empathy, and without judgement Communicating in a polite and kind manner Interacting with clients in non‐stigmatising ways (e.g., not questioning patients about their reasons for seeking care, choosing words that do not moralise or judge) Increasing attention to improving technical abortion provision skills and medical professionalism

#### 3.2.1 Increasing Abortion‐Related Communication With Colleagues

Interviewees in different legal contexts described observing that VCAT enabled participants to speak more openly and explicitly about the stigmatised topic of abortion with colleagues. As one said, it created a safe space ‘to allow the word abortion to like escape their lips, and to just feel okay with that’ (ID09, facilitator‐curriculum designer, medical education and training, United States). In some places, they noted that this ability to speak about abortion was new: ‘[The] silence is broken, and then they feel more power to talk […]. Sometimes before the VCAT, it didn't happen’ (ID14, facilitator‐participant, INGO, Latin America).

Interviewees also said that VCAT could help participants develop the ability to communicate about their needs and preferences in relation to abortion provision with colleagues, to align with differing levels of comfort within the workforce. In the words of one VCAT facilitator, ‘[VCAT can foster] ‘a willingness to […] say, I'm willing to do this part of abortion care. But could you just keep me out of this [other part]?’ (ID01, facilitator‐participant, community organisation, Australia).

These forms of communication in healthcare teams can lay the groundwork for person‐centred care provision.

#### 3.2.2 Reducing Delays and Obstruction of Abortion Care

Two interviewees, in legal settings where abortion is criminalised or highly restricted, and highly stigmatised, said they had observed that VCAT reduced the reporting of alleged abortion seekers to authorities by healthcare providers. One of them stated, ‘the reports to authority decrease a lot […] after a VCAT’ (ID14, facilitator‐participant, INGO, Latin America). Another reflected how:

You're moving people up the sympathy spectrum. So you know, if somebody truly feels morally opposed [to providing abortion care] themselves, [VCAT is] getting them to a space of ‘okay, but I can at least not report [to the authorities] this person that I think you know, took mifepristone. I could at least provide information [to the abortion seeker]. I could at least provide a referral.’

(ID09, facilitator‐curriculum designer, medical education and training, United States)

Interviewees across contexts also hypothesised that VCAT could help participants recognise, and decide to reduce, their obstructive behaviours. As one said, ‘[VCAT] helped people understand the impacts of delays and potentially reduced their motivation to delay care’ (ID01, facilitator‐participant, community organisation, Australia). Another noted that ‘it just kind of allows people to, at minimum, not be a barrier in ways that, like, they might not even have known that they were being’ (ID03, facilitator‐curriculum designer, INGO, United States and multi‐regional).

Some interviewees related this newfound motivation to reduce obstruction to the ways in which VCAT participants become aware of the harms of their past actions. For example, one interviewee described a healthcare provider who realised her past behaviours had been obstructive and verbalised her intention to shift this behaviour. ‘[After VCAT she] said that she regrets all the behaviour she projected towards her patients. And she promised to us that […] she will respect the right of her patients to obtain abortion’ (ID05, facilitator, community organisation, Nepal). The interviewee was, however, unable to ascertain if this VCAT participant had ultimately shifted her behaviour.

#### 3.2.3 Enabling Abortion Seekers to Access Abortion Care

Interviewees said that, after VCAT, they saw providers who did not provide abortion care start to take actions to enable access – potentially reflecting their increasing acceptance of, and decreasing stigma around, abortion. In a context of a public health organisation supporting abortion access in the face of legal restrictions, one facilitator said they had observed their team begin to actively help clients after VCAT. ‘Teams [began] actually com[ing] to me and then finding their own way [to help someone get care. This] meant, like, [they] want to do this’ (ID13, facilitator, INGO and local healthcare service).

Some interviewees also described observing healthcare providers begin to tolerate feelings of discomfort to balance their own beliefs with patient rights to access care. For example, a VCAT facilitator and a VCAT participant, who had been in the same session, both described observing a conscientious objector express a newfound willingness to set up the examination room for abortion procedures. They described an ‘extraordinary’ shift in a declared objector, who after VCAT reportedly told their colleagues that ‘I can probably do that [set up the room], even if I'm against termination. I can still do that for my colleagues and for the woman’ (ID12, participant, local healthcare service delivery, Australia).

Interviewees across contexts said they hypothesised that VCAT resulted in healthcare providers refering for abortion care when they hadn't done so before. One described a VCAT participant telling them, ‘I realised that the work I'm doing is not killing babies […]. It's about saving the life of the pregnant woman. So I helped them, and I've directed them to the clinic where they can get rid of the pregnancy’ (ID11, facilitator, INGO, Sierra Leone).

These findings demonstrate that, even when a healthcare provider does not directly provide abcare, VCAT may encourage them to take actions to support access for abortion seekers.

#### 3.2.4 Expanding Own Abortion Provision

Two facilitators hypothesised that healthcare providers who previously had not provided abortion care might start to provide the service after VCAT. As one noted:

There were some people that would come out of it [VCAT], who usually, I would say, came in support[ive of abortion] but [feeling that they] were in the minority. And [they] didn't feel like they could talk about [abortion], and were worried about talking about it. Who went from being supportive to being providers [of abortion].

(ID13, facilitator, INGO and local healthcare service)

Interviewees hypothesised that VCAT participants could become more willing to provide care for patients seeking more highly stigmatised forms of abortion care – in particular, abortion at later gestational ages and abortion for people seen as having ‘too many’ abortions.

One facilitator, who was also an abortion provider, said their own clinical practice had changed from their VCAT experience. ‘Since I've done [VCAT], I've kind of gone a little bit further in the limits in the gestation that I used to do surgically. […] It seems to create a kind of a space for acceptance of that’ (ID13, facilitator, INGO and local healthcare service). The interviewee connected this shift to how VCAT built healthcare providers' understanding of why someone might seek abortion later in pregnancy, which can help destigmatise that type of abortion care:

[VCAT is] bringing out some of those [abortion] stories in ways that people relate to and [making them] think, ‘Oh, yeah, you know, that could be me’. Or ‘I can see that, I can see the real story. I can see this situation happening, and I can have empathy for that.’ I think that really helps […] hopefully not to stigmatise in that care. Then to take people where they are, not where you think they should be. […] [For example, you can avoid wondering] ‘it's 22 weeks [of pregnancy already]. Why don't you come before now?’

(ID13, facilitator, INGO and local healthcare service)

Another interviewee noted that VCAT was particularly useful to help participants reflect on their feelings about abortion in later gestation without the ‘justification’ of a foetal condition. ‘[VCAT] just provides a bit more context as to why people may seek terminations over 20 weeks [or] over 24 weeks that aren't [for medical abnormalities]’ (ID04, facilitator‐participant, local healthcare service delivery, Australia).

Interviewees also mentioned that VCAT could make healthcare providers more willing to provide care to abortion seekers who had ‘too many’ abortions. One facilitator described observing a shift in the attitudes of a midwife who participated in VCAT:

[A midwife] was like, ‘I'm fine with abortion, but when they come back for their 4th or 5th abortion, what am I meant to do. I'm not willing to do it’. This is probably the most common area we see stigma among practitioners in Australia, and [the participants who said that] completely changed their tune throughout the course of the [VCAT] training.

(ID01, facilitator‐participant, community organisation, Australia)

This sentiment was corroborated by a VCAT participant, who reflected on how VCAT affected her own perceptions of patients who have more than one abortion:

I suppose it maybe just normalised, normalised the situations [of] maybe people who […] have multiple counts of abortion care. […] Rather than […] demonising […]. That was probably quite a moment for me in that workshop.

(ID10, participant, local healthcare service delivery, Australia)

#### 3.2.5 Engaging in High‐Quality, Non‐Judgemental Care

Interviewees hypothesised that VCAT might reduce stigmatising behaviour by making providers aware of the judgement they might be holding. One facilitator said that VCAT was designed to help providers ‘assess and realise their own biases and how to work around them […]. It can lead to a nonjudgmental, empathic high quality, abortion service’ (ID08, facilitator‐curriculum designer, community organisation, Ghana). Another similarly noted:

I think it [VCAT] makes us take more ownership over our behaviour and the outcome of our behaviour. […] It really makes people think about actually, my values are this, and I don't want to cause harm, and I don't want to cause stigma. I didn't realise that what I'm doing was causing stigma.

(ID15, facilitator, community organisation, Australia)

Corroborating this sentiment, one VCAT participant described how VCAT created a space for them and their healthcare providing colleagues to reflect about how their abortion‐related judgements and beliefs might affect the care they provide.

I'm human. I also judge people. Not that I let that affect me professionally in how I show it. But I'm aware of it internally. So, I think it [VCAT] provided a good opportunity to reflect with colleagues.

(ID10, participant, local healthcare service delivery, Australia)

Some facilitators recounted conversations with participants who, after VCAT, articulated their intentions to be less judgemental. Several described religious participants who expressed a desire to ‘be more compassionate towards people that I see’ and ‘reserve judgement’ (ID17, facilitator, INGO, United States).

Interviewees also hypothesised that VCAT could motivate providers to ensure high‐quality care. As one expressed, ‘if you believe someone's deserving, you will give them good quality care’ (ID01, facilitator‐participant, community organisation, Australia). One facilitator noted that VCAT supports ‘equal care to people of whatever sex, religion, colour, whatever. So [it shows that] your own beliefs […] shouldn't influence what kind of service you should provide’ (ID08, facilitator‐curriculum designer, community organisation, Ghana). Another similarly noted that VCAT helped providers see abortion as a service to be provided ‘like any other’ (ID16, facilitator‐participant, local healthcare service delivery, Kenya).

One interviewee suggested that VCAT can encourage providers to bring ‘more attention to upgrading their [abortion care] skills. Because they now realise, yes, I need to be more professional, and for you to be more professional, your skills should be enhanced’ (ID11, facilitator, INGO, Sierra Leone). Another noted that VCAT could be complemented by technical skill development activities and practice to reinforce behaviour change:

[Our broader training beyond VCAT] was also doing a lot of role plays and a lot of, like, demonstrations of what, at that time, woman‐centred, or client‐centred or person‐centred care looks like. And then having people practice and give feedback in a simulated setting, and then doing it in the clinic setting, and then having feedback. So just, you know, practice and feedback sessions are incredibly important for learning and reinforcing behaviour change.

(ID07, facilitator‐curriculum designer, INGO, multi‐regional)

These observations illustrate potential strategies that can complement VCAT to strengthen its potential contributions to person‐centred behaviours in abortion care.

### 3.3 Empathy as a Mechanism to Reduce Stigma and Increase Person‐Centred Care

The theme of empathy was prominent across the analysis and became a key organising concept for the findings. All interviewees described empathy as a central mechanism through which VCAT seemed to contribute to behaviour change and destigmatisation. In the words of one interviewee, ‘empathy is like the portal to the change I see happening in the room’ (ID03, facilitator‐curriculum designer, INGO, United States and multi‐regional). They described three main forms of empathy that they believed VCAT influenced in healthcare providers in ways that support behaviour change: a) humanising abortion seekers; b) building understanding of the reasons people seek abortions; and c) centring the needs of patients. Each category is described in more detail in the following sections, with example quotes.

#### 3.3.1 Humanising Abortion Seekers

Interviewees hypothesised that one of the ways that VCAT built empathy towards abortion seekers was through humanising their behaviour. One facilitator said that ‘a lot of the VCAT is […] helping to see the [abortion patient] as a person. And so, I do think there's a lot of destigmatization that goes on in that’ (ID13, facilitator, INGO and local healthcare service). Another facilitator described ‘deeply Christian folks […] saying, this is the first time I've, like, had a conversation about this and saw [abortion seekers are] just another human’ (ID03, facilitator‐curriculum designer, INGO, United States and multi‐regional).

An interviewee who had designed VCAT activities described the purposeful inclusion of empathy‐building within VCAT by helping providers see what they have in common with abortion seekers:

VCAT exercises are designed to invoke empathy very intentionally […] and also to surface double standards and hypocrisy that people carry often unconsciously. […] Why do we believe that we would make responsible decisions, but that people having abortions […] aren't? […]. The whole purpose was to give people an ‘aha’ moment to realise that they are holding these hypocrisies.

(ID07, facilitator‐curriculum designer, INGO, multi‐regional)

Corroborating the idea that VCAT helps humanise abortion seekers, a participant described how VCAT increased participants' compassion for the life circumstances of abortion seekersand helped them recognise the similarities between abortion seekers and other types of clients.:

You accept that we live in an imperfect world, and everyone's chronic disease isn't optimally managed. And similarly, you know, people perhaps don't always make optimal contraceptive choices and then find themselves in situations where they need to access abortion care. […] I think [after VCAT] there was […] more compassion for the circumstances that people find themselves in. Just a little bit more understanding of those other complexities or those other issues we're not necessarily hearing about, but might be there [from our patients].

(ID10, participant, local healthcare service delivery, Australia)

#### 3.3.2 The Reasons Why People Seek Abortion Care

One form of empathy instigated by VCAT is compassion for the many reasons people might seek abortion care. As one facilitator said, ‘when we break down the reasons why […], they [VCAT participants] really start to expand their understanding not from a heady way, but like in a really embodied, empathic, heart‐driven way’ (ID03, facilitator‐curriculum designer, INGO, United States and multi‐regional). A facilitator‐participant similarly noted, ‘I think that understanding some of the context as to why a pregnant person may seek an abortion over 24 weeks, actually, you know, puts things into perspective a little bit’ (ID04, facilitator‐participant, local healthcare service delivery, Australia). Corroborating this point, a VCAT participant said that VCAT can build awareness of ‘a myriad of reasons why someone is seeking this [service]. […] I think [VCAT] can change the lens that they [providers] look at these [abortion seeking] patients through’ (ID12, participant, local healthcare service delivery, Australia).

Interviewees described how VCAT also builds an understanding of the importance of providing abortion care regardless of reason. This is important because ‘you actually don't need a reason [to justify having an abortion], but for a lot of staff to provide compassionate care, they need to know the reasons’ (ID04, facilitator‐participant, local healthcare service delivery, Australia). Another interviewee hypothesised that VCAT helps participants realise that they ‘can't ever know the full story of why this person is choosing that’ (ID03, facilitator‐curriculum designer, INGO, United States and multi‐regional). Another similarly said:

I think [VCAT] can help build more compassion, and to actually undo what people view an abortion to be. Because […] there will always be experiences that you don't think of. Like, actually, I don't know what you're going through, but I know you're here for care.

(ID06, facilitator, community organisation, United Kingdom and multi‐regional)

#### 3.3.3 Centring the Needs of Abortion Seekers

Some interviewees hypothesised that VCAT helped providers consider and prioritise the specific needs and circumstances of their abortion seeking patients. As one said, ‘VCAT [has] a mechanism to think through those sticky moments when it's ultimately not about what you think as the provider, but rather what the patient needs. And so recentring the patient’ (ID02, facilitator, medical education and training, United States). Another facilitator similarly said that ‘VCAT helps [providers] be empathic, [for example] thinking about young girls coming in for post‐abortion care, […] being centred to their particular needs’ (ID14, facilitator‐participant, INGO, Latin America).

Several interviewees explicitly linked the empathy built through VCAT to potential provision of non‐judgemental care. One medical educator said, ‘If we don't talk about how to be empathetic and the importance of empathy and the ultimate goals of providing nonjudgmental, patient‐centred care then, like, sometimes it won't happen’ (ID02, facilitator, medical education and training, United States). Another interviewee explained:

When you internalise and empathise with the [people] who are in difficult circumstances and who want [an] abortion, then your behaviour will automatically change, I believe. Because you will not see a patient as a mere patient. You will see the whole story.

(ID05, facilitator, community organisation, Nepal)

#### 3.3.4 Discussion

Abortion Values Clarification and Attitude Transformation (VCAT) is used around the world, yet we lack evidence about whether and how it can be effectively harnessed to target harmful behaviours in the health system [28]. This study helps bridge this gap, drawing on interviews with those who have designed, facilitated and participated in VCAT to build hypotheses about (a) the specific behaviours VCAT may influence and (b) the mechanisms through which it might do so. Importantly, this study sought to develop hypotheses about how VCAT might influence behaviour, rather than to evaluate VCAT or measure behaviour change. Analysis drew primarily on the perspective of facilitators and designers of VCAT, who are experienced with the approach but also likely to have positive perceptions of it based on their professional involvement. We used the perspectives of our few VCAT participant interviewees to corroborate or deepen hypotheses expressed by facilitators, but this perspective is limited due to challenges recruiting VCAT participants.

Our analysis contributes to the literature about VCAT in two main ways. Firstly, our findings generate nuanced evidence about the ways in which empathy may promote behaviour change, extending the idea that VCAT builds empathy towards abortion seekers articulated in Ipas' conceptual model [34]. The specific behaviours identified in this study appear to be grounded in the development of three specific types of empathy through VCAT: empathy for abortion seekers, for the many reasons they may seek care, and for their needs and preferences on their pathway to care. By promoting these forms of empathy, VCAT appears to create a sense of abortion as a worthy type of healthcare and abortion seekers as worthy of receiving care, which may motivate behaviour change in healthcare providers. Secondly, our analysis generated hypotheses about specific behaviours that appear to be influenced by VCAT (Table 2), which can be measured in future evaluations to assess whether the approach contributes to behaviour change. For example, these behaviours could be measured by: documenting stories of behaviour change; collecting self‐reported data about changes in person‐centred care behaviours from providers who have received VCAT; using direct observation of provider interactions with mystery clients; collecting data about experiences in care from the patients of healthcare providers after VCAT; or comparing disaggregated service delivery statistics (e.g., by gestational age at the time of abortion or by patient type) before and after VCAT. These approaches could help address an evidence gap, as VCAT evaluations rarely examine or measure behaviour change [27, 28, 38, 40, 41].

**Figure 1:**
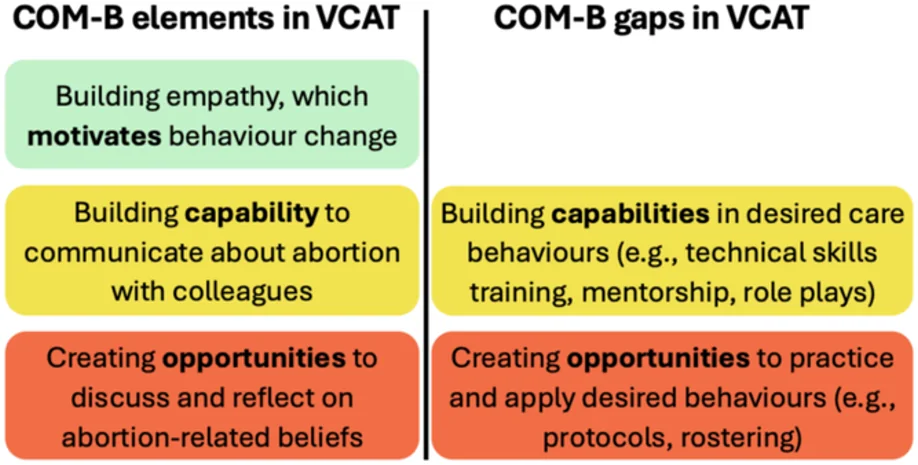
Mapping VCAT to the COM‐B model.

Our study is novel in its mapping of VCAT onto key concepts in behaviour change theory: capability, opportunity and motivation [42]. Our findings highlight the ways that VCAT might **motivate** behaviour change in healthcare providers. For example, we show that a new understanding of the impacts of delayed care might motivate a healthcare provider to reduce obstruction; in some cases VCAT participants articulated their intentions to change their obstructive behaviour (3.2.2) or to enable access to care, even in highly restrictive legal contexts (3.2.3). We also show that VCAT may motivate providers to expand the types of abortion care they provide (3.2.4) or to engage in skill development to ensure they are ready to provide high‐quality care (3.2.5). We demonstrate how VCAT can build providers' **capability** to increase their communication about abortion in the workplace by creating **opportunities** for VCAT participants to practice discussing abortion with their colleagues (3.2.1) and to reflect with colleagues about the ways that abortion‐related judgements that might influence care delivery (3.2.5). Importantly, however, our findings highlight some of the gaps in VCAT that may limit its effects on behaviour change: the need to build **capability** in person‐centred abortion service provision skills and then create **opportunities** in the workplace to practice and apply these skills (3.2.5). This suggests that VCAT may benefit from complementary strategies that build healthcare provider capabilities in person‐centred clinical and communication skills, and ensure sufficient opportunities to practice and consolidate these [Figure 1]. This reflects evidence that a combination of capability, opportunity and motivation is more likely to foster behaviour change than an intervention with just one of these elements [42]. Taken together, our findings can inform the refinement of VCAT and development of complementary interventions that can encourage person‐centred abortion care provision through a combination of motivation, skills‐development and opportunity creation, as has been done in contraception‐related interventions [46].

This study also examines the interrelationships between empathy, stigma and person‐centredness – key themes arising from our analysis. For example, when more empathic healthcare providers reduce their obstruction or begin to facilitate supportive care (a key domain of person‐centred care), they are resisting the stigmatising belief that abortion is morally wrong and/or socially unacceptable [5, 9]. When they overcome the silence around abortion at work (stigma) by speaking compassionately about abortion (empathy), they legitimise abortion care and encourage their workplace to engage in it (health facility environment domain of person‐centred care) [2, 47]. When they respect the needs and preferences of abortion seekers (empathy), they avoid judgement and discrimination (stigma) and acknowledge that abortion care should be person‐centred [12, 21]. When providers realise that abortion seekers should not have to justify their abortion or share their reasons for seeking care (empathy), they are aligning with abortion seeker preferences for stigma‐free and person‐centred care [48, 49, 50]. The academic literature rarely puts these three concepts into dialogue, instead tending to examine two at a time. While some studies have considered empathy as a key aspect of high quality person‐centred care [16, 21, 50, 51, 52], and others have described empathy as a facet of stigma reduction [27, 28, 34], this study ties these efforts together by hypothesising about empathy's role as a mechanism of stigma reduction that helps produce person‐centred care behaviours.

Our findings illustrate VCAT's capacity and flexibility to encourage behaviour change across very different sociocultural contexts, dominant forms of stigma, and legal frameworks around abortion. For example, study participants in contexts where abortion is highly legally restricted described VCAT's effective messaging about the deadly consequences of refusal and how these do not align with providers' core values of care, as documented in VCAT approaches in Asia, Africa, and Latin America [27, 28]. In contrast, participants where abortion is decriminalised said they focused VCAT on more highly stigmatised types of abortions – similar to past efforts to tailor VCAT for second trimester abortion [29] or for adolescents [34]. This is important because even where abortion is decriminalised, some types of abortion are considered more legitimate than others [53, 54, 55].

Our results also suggest that VCAT may motivate participants to move along a spectrum from opposing to supporting abortion access. The specific behaviours on the spectrum appear to reflect the local legal and socio‐cultural context. For example, in countries where abortion is highly restricted, VCAT may help discourage reporting alleged abortion seekers to the authorities – a behaviour that is not relevant in decriminalised contexts. These results align with research from Ethiopia suggesting that VCAT reframes abortion in ways that encourage reduced gatekeeping or expanded provision [28]. In doing so, VCAT is hypothetically positioned to expand person‐centred care for those who already provide care and mitigate harmful behaviours among those who do not. This can have benefits for the abortion care workforce, as increasing support for abortion access in the healthcare workforce can help reduce burnout among the limited providers who offer the service regularly [11]. It would be useful to explore how VCAT could be more explicitly harnessed to address stigmatising referral practices [56] or its potential application in the wake of legal changes, new institutional leadership, or health system expansion of abortion provision.

## 4 Strengths and Limitations

A strength of our study is its global and multi‐organisational scope, integrating perspectives of VCAT facilitators and participants who have experienced a range of VCAT manuals, designs, and approaches in diverse settings. The findings suggest that, regardless of legal and socio‐cultural setting, tailored VCAT can support empathy‐building and destigmatisation to help promote access and person‐centred care. This adds value to the literature to date, which has largely examined one VCAT implementation (e.g., one organisation following a specific manual in one or multiple locations).

This study also has several limitations. First, the small number of interviewees who were VCAT participants means our data include only a minimal perspective of the intended intervention recipients. Future studies should seek to measure whether VCAT influences behaviours in healthcare providers; the hypotheses generated in this study identifying specific behaviours that VCAT may influence can inform such studies. Second, most study participants were facilitators and designers of VCAT, who may be personally and professionally invested in VCAT's effectiveness. The interviewer addressed this risk of confirmation bias by clarifying that the study sought to build hypotheses based on participants' intimate understanding of VCAT, rather than evaluating VCAT. Third, interviewees may have believed that the interviewer hoped to hear about positive or successful facets of VCAT. To minimise the risk of social desirability bias, the interviewer informed all participants that they had never designed or facilitated VCAT and were not affiliated with a VCAT‐providing organisation. Further, the interview guide included prompts about gaps in VCAT and asked for interviewees to share information about what VCAT was not designed to achieve. Fourth, we only conducted interviews in English, potentially limiting participant perspectives. However, the global reach of our study and range of professional backgrounds of participants help ensure diverse perspectives are centred. Finally, it is important to acknowledge that VCAT alone is unlikely to lead to sustained behaviour change, as healthcare provider behaviours are shaped within their social, institutional and political environments. We have published a complementary analysis, beyond the scope of this paper, to examine the institutional and structural factors that influence VCAT implementation [57].

## 5 Conclusion

This qualitative study helps fill an evidence gap about whether and how abortion VCAT might move beyond shifting knowledge, attitudes and intended behaviours to actually changing healthcare providers' behaviours. Our findings show that VCAT could hypothetically reduce abortion obstruction and increase communication about abortion in the workplace and engagement in person‐centred interactions with abortion seekers. Our results suggest that the interrelated processes of destigmatisation and empathy‐building promoted by VCAT can motivate changes in these behaviours in health workers across contexts, legal settings, and modalities of intervention delivery. Future studies, using a range of approaches to assess if the specific behaviours identified in this study do indeed change after VCAT, are needed.

Based on mapping VCAT to the COM‐B model of behaviour change, our study also identifies VCAT's potential gaps that may limit its effectiveness at influencing behaviour. Alongside the motivation generated by VCAT, complementary strategies are likely needed to support healthcare providers to develop capabilities in person‐centred communication, referral and care, and to create opportunities to practice and apply these skills. These findings contribute to the literature about how to encourage stigma reduction and person‐centred abortion care, and can inform future work to refine and evaluate VCAT and similar interventions globally – helping reduce gendered inequities in access to care.

## Author Contributions

**Shelly Makleff:** conceptualisation, methodology, formal analysis, supervision, writing – original draft, writing – review and editing, project administration. **Carrie Van Rensburg:** data curation, formal analysis, writing – review and editing. **Bronwen Merner:** writing – review and editing. **Alexandra Wollum:** writing – review and editing. **Louise Keogh:** methodology, writing – review and editing.

## Ethics Statement

Approval was granted by The University of Melbourne Human Ethics Committee (ID 30693). Written or verbal informed consent was obtained from all participants.

## Conflicts of Interest

The authors declare no conflicts of interest.

## Data Availability

Research data are not shared. The datasets generated and analysed during the current study are not publicly available due to the sensitive nature of the data collected but are available from the corresponding author on reasonable request.
